# Time from Final Oncologist Visit to Death and Palliative Systemic Treatment Use Near the End of Life in Heavily Pretreated Patients with Luminal Breast Cancer

**DOI:** 10.3390/jcm13226739

**Published:** 2024-11-08

**Authors:** Mirosława Püsküllüoğlu, Marek Ziobro, Małgorzata Pieniążek, Renata Pacholczak-Madej, Sebastian Ochenduszko, Iwona Godek, Agata Adamkiewicz-Piejko, Aleksandra Grela-Wojewoda

**Affiliations:** 1 Department of Clinical Oncology, Maria Sklodowska-Curie National Research Institute of Oncology, Kraków Branch, 31-115 Krakow, Poland; marek.ziobro@krakow.nio.gov.pl (M.Z.);; 2 Department of Oncology, Wrocław Medical University, 50-367 Wroclaw, Poland; 3 Lower Silesian Comprehensive Cancer Center, 53-413 Wroclaw, Poland; 4 Department of Anatomy, Jagiellonian University Medical College, 33-332 Krakow, Poland; renata.pacholczak@uj.edu.pl; 5 Department of Gynecological Oncology, Maria Sklodowska-Curie National Research Institute of Oncology, Krakow Branch, 31-115 Krakow, Poland; 6 Department of Chemotherapy, The District Hospital, 34-200 Sucha Beskidzka, Poland; 7 Hospital Universitario Doctor Peset, 46017 Valencia, Spain; 8 Department of Palliative Care, Urban Center for Care of Elderly and Chronically Disabled Persons, 30-663 Krakow, Poland

**Keywords:** breast cancer, metastases, systemic treatment, end-of-life care, palliative care, advance care planning, death

## Abstract

**Background**: Palliative care must be tailored for patients with extended disease trajectories, such as those with hormone receptor-positive, Human Epidermal Growth Factor Receptor 2 (HER2)-negative advanced breast cancer (ABC), including the appropriate timing of discontinuing treatment. This study aimed to assess the interval between the last oncologist visit and death and the application of systemic treatment near the end of life in this patient population. **Methods**: This retrospective study included patients with luminal ABC who received at least two lines of palliative systemic treatment at the National Research Institute of Oncology in Poland, and died between November 2020 and March 2024. **Results**: Seventy-six women, with a median age 62.8 years (range: 35.3–91.5), were included. The median number of prior palliative systemic treatment lines was three (range: 2–6). At their last recorded oncologist visit, 75% of the patients were receiving active treatment (53% with hormonal therapy and 22% with chemotherapy). Only 25% were under continuous palliative care at this visit. Treatment was administered within the last month of life to 53% of the patients. The median duration from the last oncologist visit to death was 23 days (range: 0–408). The duration of this time interval was only associated with the performance status at the last visit (*p* < 0.05). **Conclusions**: Oncologists frequently delay the recognition of the need to discontinue systemic therapy. Patients with luminal HER2-negative ABC may be offered numerous effective lines of systemic treatment, complicating this decision further. Implementing clearer guidelines for end-of-life care for this group and providing proper training for healthcare providers is essential.

## 1. Introduction

Globally, breast cancer (BC) is the foremost cause of death among women [1]. The classification of BC is based on the expression of estrogen receptors (ERs) and progesterone receptors (PRs), the HER2 status, and the Ki-67 levels, resulting in subtypes such as luminal A, luminal B, HER2-positive, and basal-like, with triple-negative BC being the most prevalent within the basal-like group [2]. Luminal breast cancers, characterized by ER/PR positivity and HER2 negativity, typically have more favorable prognoses, even when metastatic. Recent advancements have been made in the systemic treatment of patients with hormone receptor (HR)-positive, HER2-negative advanced breast cancers (ABCs) allowing for numerous effective treatment lines [3,4].

Treatment options for luminal HER2-negative metastatic breast cancer encompass a range of therapeutic approaches. These include cyclin-dependent kinase 4/6 (CDK4/6) inhibitors in combination with endocrine therapy [5,6,7,8,9,10], alpelisib paired with fulvestrant for phosphatidylinositol-4,5-bisphosphate 3-kinase catalytic subunit alpha (PIK3CA)-mutated cases [11,12], exemestane alongside everolimus [13], and elacestrant as a selective estrogen receptor degrader, especially in patients with an estrogen receptor 1 gene mutation [14]. Additionally, poly ADP-ribose polymerase (PARP) inhibitors are effective for patients with *BReast CAncer* gene (*BRCA1/2*) mutations [15,16]. Trastuzumab deruxtecan showed efficacy in HER2-low metastatic breast cancer [17], while sacituzumab govitecan is recommended in further therapy lines [18]. When hormonal and targeted therapies are exhausted or in cases of visceral crisis, chemotherapy remains an option. Overall, these patients receive numerous treatment lines in the palliative setting [3,4].

As treatment progresses and options narrow, disease-modifying therapies often lose their effectiveness and may cause more harm than good. Consequently, palliative and supportive care become the primary focus. In the early stages of metastatic cancer, extending survival may be prioritized over patient comfort. However, as the disease advances, the emphasis shifts towards ensuring comfort and maintaining function [19,20].

Palliative care plays a crucial role in the management of patients with ABC. As ABC progresses, the symptom burden and psychosocial needs become increasingly complex, often necessitating a comprehensive approach to care that includes both symptom management and end-of-life (EOL) planning. The early integration of palliative care is associated with significant benefits, including an enhanced quality of life, reduced symptom severity, and improved alignment of care with patients’ values and preferences [19]. Studies indicate that patients with ABC who receive timely palliative care interventions, ideally within six months of a diagnosis, are more likely to engage in EOL discussions, complete advance directives, and experience a reduction in aggressive medical interventions, such as hospitalizations and chemotherapy, in the last months of life [21]. Despite palliative care’s goal to alleviate suffering at all stages of oncological disease, including addressing treatment-related issues in breast cancer patients from diagnosis through EOL care, studies indicate that it typically begins only when disease-modifying treatments are no longer effective [20,22,23,24]. For women with ABC, it is crucial to identify when such treatment should be discontinued. Many oncologists do not apply clinical criteria to guide decisions based on therapeutic proportionality, beneficence, and non-maleficence, leading to potential therapeutic futility and a decline in patients’ quality of life [20,25]. Recent research has identified several palliative care models for ABC patients, each with unique advantages [19,21,26,27]. In the oncologist-led model, oncologists provide both cancer treatment and palliative support, fostering continuity but potentially lacking specialized PC expertise. The concurrent model, where palliative care specialists work alongside oncologists, improves the quality of life and reduces aggressive end-of-life interventions by focusing on symptom management and psychosocial support. The trigger-based model initiates PC at specific clinical milestones, ensuring timely intervention but requiring precise criteria, especially in hormone receptor-positive disease with extended survival. Lastly, the consultative model offers palliative care on an as-needed basis, typically later in the disease course, which addresses immediate needs but may delay early symptom management. Concurrent and trigger-based models are increasingly favored for their proactive, patient-centered approach [19,21,26,27].

The aim of this study was to assess the interval between the last oncologist visit and death and systemic treatment application near the end of life in patients with hormone receptor-positive, HER2-negative advanced breast cancer.

## 2. Materials and Methods

### 2.1. Patients

Patients diagnosed with ABC and treated at the National Research Institute of Oncology, Krakow Branch, who completed a minimum of two systemic treatment lines in a palliative setting (including a minimum of one that was hormonally based) and died between November 2020 and March 2024 were included in this study. Other inclusion criteria comprised individuals aged 18 years or older, demonstrating HER2-negative status, and ER or PR levels ≥ 1%. The exclusion criterion was a coexisting second malignancy. There were no limitations regarding patient sex, type of hormonal agents, targeted agents, or chemotherapy received in the past.

ER and PR status was assessed as positive if ≥1% of invasive cancer cells showed nuclear staining. Immunohistochemistry was performed to evaluate the expression of HER2 and the status was assessed as negative for a score of 0 or 1. For a score of 2, fluorescence in situ hybridization (FISH) analysis was performed to distinguish between positive and negative cases [28,29].

### 2.2. Data Collection

For this retrospective cohort study, we have gathered data from patients’ clinical records regarding age, clinical staging at diagnosis, date of diagnosis, dates for and types of treatment, and metastases dates, location and treatment; date of the last visit and date of death; histopathological data including histology, tumor grade, status of HER2, PR, ER, and Ki-67, and performance status (PS) during the last visit.

In this retrospective study, clinical data were gathered from a combination of digital and paper-based records. Data extraction occurred from April to June 2024, and was conducted by three medical oncologists (MP, AR, RPM), who manually reviewed each patient’s records, ensuring thoroughness and accuracy. Data were anonymized. Key data points, including treatment dates, clinical staging, and histopathological details, were entered into a secure digital database. To maintain data accuracy, each entry was cross-verified by one additional oncologist. Data from records spanning the period of initial diagnosis up to the last documented visit before death were included, providing a comprehensive overview of each patient’s clinical history.

### 2.3. Ethical Considerations

This study was approved by the Ethical Committee at the Maria Sklodowska-Curie National Research Institute of Oncology, Warsaw Branch, Poland (registry number 20/2024 dated 22 Feb 2024). Due to the retrospective nature of the study, the Ethical Committee waived the requirement for informed consent. All procedures and protocols complied with relevant guidelines and regulations including the Declaration of Helsinki.

### 2.4. Statistical Considerations

Mean, standard deviation, median, quartiles, and range of quantitative variables are shown. For qualitative variables, absolute and relative frequencies (% and N) are reported. The Mann–Whitney test was used for comparisons of quantitative variables between two groups, while the Kruskal–Wallis test (followed by a post hoc Dunn test) was used for three or more groups. Spearman’s correlation coefficient was applied to assess correlation between two quantitative variables.

The significance level was set to 0.05. All the analyses were conducted in R software, version 4.3.3

## 3. Results

### 3.1. Population Characteristics

76 patients were included in this study, and all were females. The median age at the last visit was 62.8 years (range: 35.3–91.5). The median time from the initial BC diagnosis to the last visit was 4.5 years with a mean of 5.9 years (range: 0.7–17.3 years). The median time from the diagnosis of metastatic disease to the last visit was 1.9 years with a mean of 2.1 years (range: 0.2–6.9 years). Table 1 presents the clinicopathological characteristic of the population.

### 3.2. Previous Treatment Received

The patients received a median of three prior lines of any palliative systemic treatment (range 2–6). The data regarding the previously applied therapies are presented in Table 2.

### 3.3. Treatment Received During the Last Visit and Palliative Systemic Treatment Use Near the End of Life

Anticancer systemic treatment was administered within the last month of life to 40 patients (52.6%), and 57 patients (75%) continued active systemic treatment during and/or after the last visit.

Nineteen patients (25%) were under palliative care support (house hospice or palliative outpatient care) before the last visit. On the day of that last visit, a further 21 patients (27.6%) were referred to receive such support and 2 patients were admitted to the oncology ward and died during that hospitalization due to disease progression. They were both undergoing active treatment. Table 3 summarizes the data regarding the treatment received on the last visit and the palliative care applied before the last visit.

### 3.4. Time from the Last Oncologist Visit to Death

The median duration from the last oncologist visit to death was 23 days (range: 0–408). Table 4 presents the details regarding this time interval.

The length of this time interval was solely associated with the patients’ PS at the last visit, with *p* < 0.001 and Spearman’s rank correlation coefficient being −0.72, indicating that the patients with a higher PS presented with a shorter time interval. This was not associated with the age, initial stage, tumor grade, ER/PR or HER2 expression level, Ki67 status, presence of visceral or brain metastases, type of previously received treatment, or number of treatment lines (all *p* > 0.05; see Supplementary Materials Tables S1–S20.).

## 4. Discussion

It is concerning that nearly one-quarter of the patients received chemotherapy during their EOL period, a practice that should be avoided [30]. When hormonal therapy is taken into consideration, this proportion was doubled. However, the issue of overtreatment and delayed referrals to palliative care is reported globally, not only in Poland, according to recent reports [31]. The implementation of palliative care and EOL decision-making practices varies significantly due to differing legislative frameworks worldwide. Some countries, like Japan, lack specific laws, embedding palliative care provisions within broader cancer control regulations. This has led to inconsistent access and reluctance among clinicians to cease cancer treatments near EOL due to potential malpractice concerns [32]. In contrast, Taiwan and South Korea have established clearer palliative care legislation [32]. Nevertheless, even among countries with palliative care legislation, discrepancies exist in the levels of implementation, with some guidelines largely attached to cancer-specific policies rather than comprehensive palliative frameworks. In Europe, the integration of palliative care faces similar inconsistencies. Although the European Union has encouraged universal palliative care integration, many healthcare systems prioritize curative over palliative measures, partly due to fragmented national policies and varying adherence to international standards like those from ASCO or ESMO [33]. This regulatory diversity underscores the need for unified global and national policies that support evidence-based palliative care, allowing clinicians to make EOL care decisions driven by patient needs rather than legal uncertainties [34].

In a study by Beaudet and colleagues regarding patients with metastatic non-small cell lung cancer, a similar proportion of the patients received anticancer treatment within the last month of life [35], as in our study. Considering the long-established ASCO guidelines, which do not recommend further antitumor therapy for most patients with solid metastatic tumors after the failure of third-line systemic treatment—justifying this with the very low likelihood of treatment success and the high probability of toxicity—the rationale for such practice should be examined also for populations with a longer expected overall survival (OS) and multiple available treatment options, like in this case [36]. In some populations, existing discrepancies between the lack of evidence for further treatment and routine clinical practice suggest that this decision is driven not by solid benefits from anticancer therapy, but by the desire to help patients, as well as the inability of patients, families, and their oncologists to stop the treatment [31]. Our patients have received a median of three systemic therapy lines. On the one hand, this is a heavily pretreated population and one may predict that any further systemic line may bring limited benefit, still with the expected treatment side effects; on the other hand, current phase 3 clinical trials have shown significant benefit with modern therapies [17,18]. In the case of patients with HER2-negative luminal ABC, even those treated with multiple lines of systemic therapy, attention must be paid to possible variations in clinical management arising from the latest clinical trial results involving antibody–drug conjugates (ADCs), e.g., sacituzumab govitecan, trastuzumab deruxtecan, or datopotamab deruxtecan. In this setting, ADCs showed a better efficacy than that of the previous standard of care (i.e., chemotherapy). In the TROPiCS-02 trial, nearly 60% of the patients in both arms received three or more lines of chemotherapy, with 58% in the sacituzumab govitecan arm and 56% in the control arm [37]. In the DESTINY-Breast04 study, over 60% of the patients in both the trastuzumab deruxtecan arm and the control arm received more than three lines of systemic treatment [38]. Despite the heavily pretreated nature of the study populations, both studies demonstrated a longer progression-free survival (PFS) and OS for the patients receiving ADCs [37,38]. In a phase I study, datopotamab deruxtecan showed promising activity in pretreated HER2-negative luminal ABC [39].

The possibility of using ADCs in later lines makes the decision of the end of systematic therapy even more complicated. The ASCO and European Society for Medical Oncology (ESMO) guidelines highlight the importance of the performance status (PS) according to the Eastern Cooperative Oncology Group (ECOG) [30,31]. A poor PS is stated as level 3 and 4. This definition is based on limited self-care and confinement to a bed or chair for more than 50% of the patient’s waking hours [40]. A poor PS was found to be a predictor of poor survival, reduced response, and worsened toxicity from chemotherapy [41]. In our study, only the PS was associated with the time from the last oncological visit to patient death, suggesting it is a valuable factor in the treatment continuation decision-making process. In our previous publication, we showed the PS to be the only factor that predicted the response to cisplatin monotherapy in HER2-negative ABC patients with hepatic visceral crisis [42]. Patients with a poor PS are excluded from clinical trials; hence, the benefit of different types of systematic treatment in this population is not established.

The selected studies identified various factors associated with the increased use of systemic treatment at the EOL. These factors include the histology of the cancer, absence of a palliative care unit within the cancer center, sex, younger age, lower levels of comorbidity, higher levels of patient education, and even the younger age of the treating oncologist [31,43].

Patients with luminal HER2-negative ABC generally have a longer OS compared to that of other populations receiving palliative care and can receive multiple effective lines of systemic therapy. Although the interval between the final oncology visit and death is brief in our study, the presence of viable treatment options, including targeted therapies beyond the third line, indicates that this should not be automatically perceived as negative or in need of alteration.

The breakthrough research results for early palliative care (EPC) were published over a decade ago [44]. The study demonstrated that patients with metastatic non-small cell lung cancer undergoing EPC had a better quality of life compared to patients who received exclusive cancer treatment. Moreover, symptoms of depression were observed less frequently, and aggressive medical procedures at the EOL were also less commonly used in this group. Despite this, the median OS rate was significantly higher [44]. Two years after the publication of that study, the ASCO established EPC as a standard for metastatic cancer [45]. One might expect that, given these data and recommendations, EPC would be integrated into routine clinical practice globally. Nevertheless, our study reveals that the integration of EPC and conventional treatment is infrequent. Only 25% of our patients were receiving continuous palliative care at their final visit, and 28% were referred to palliative care for the first time. Despite the documented benefits, palliative care is frequently underutilized or introduced late in the management of life-threatening illnesses like cancer in numerous countries [24]. Numerous studies associate palliative care and the extended duration of palliative support with improved EOL quality indicators and even reduced costs in patients with various types of malignancies [46,47,48]. EPC started even during active oncologic treatment not only addresses physical symptoms such as pain, fatigue, and dyspnea, but also provides psychological support that mitigates anxiety, depression, and emotional distress, which are common in ABC [21]. Furthermore, systematic reviews reveal that EPC integration correlates with decreased rates of intensive EOL interventions, including intensive care unit admissions and last-minute hospitalizations, and instead supports a more managed transition to hospice and home-based care when appropriate [27]. Integrated PC models, where palliative care specialists work alongside oncologists, have shown success in facilitating early referrals and enhancing care continuity, allowing patients to benefit from both disease-modifying and supportive therapies throughout their cancer journey [26].

### Study Limitations

This study has notable limitations that merit consideration. The retrospective nature of the research introduces a potential selection and recall bias, which may compromise the accuracy of the data obtained from patient records. Furthermore, the single-institution setting may restrict the generalizability of the results to broader populations. Although the sample size was sufficient for initial analysis, it may not be large enough to identify smaller effect sizes or subtle associations. We excluded the patients treated in earlier years due to the rapidly changing treatment landscape in this setting.

Additionally, excluding patients with coexisting second malignancies, while necessary for maintaining a homogeneous study population, might overlook the complexities associated with managing patients with multiple health conditions. The diversity in treatment regimens, including various hormonal therapies, targeted agents, and chemotherapy protocols, could also introduce confounding variables that were not fully controlled for in the analysis.

## 5. Conclusions

The decision to discontinue anticancer treatment is one of the most challenging tasks for patients, their families, and experienced healthcare providers. Patients with luminal HER2-negative ABC have survival durations measured in years and may be offered numerous effective lines of systemic treatment, significantly more than other populations of palliatively treated cancer patients. This abundance of options further complicates the decision-making process. While the time from the last oncology visit to death appears very short, the availability of effective treatments, including targeted therapies as a third line and beyond, suggests that this situation should not be viewed as unequivocally negative or necessarily requiring change. However, the fact that over 50% of patients receive treatment during the last month of life is concerning.

This study highlights the need for evidence-based guidelines to inform EOL care in patients with metastatic disease and an expected longer survival with numerous available treatment options such as luminal HER2-negative ABC. Future prospective studies should validate the PS and other factors as predictors of EOL outcomes, potentially establishing standardized protocols. Clinically, incorporating such protocols into EOL guidelines would enable timely, objective treatment decisions, optimizing the quality of life by minimizing late-stage aggressive interventions. The early integration of palliative care, particularly in patients with a poor PS, may improve symptom management, align treatment with patient preferences, and reduce unnecessary interventions. Novel therapies, including antibody–drug conjugates, further necessitate research into optimal cessation points to prevent overtreatment. Additionally, proper training for healthcare providers should be implemented.

## Figures and Tables

**Table 1:** Clinicopathological characteristic of the population.

Parameter		Total (N = 76)
Stage at diagnosis *	IV	19 (25.0%)
	I–III	57 (75.0%)
Grade	G1	5 (23.7%)
	G2	45 (59.2%)
	G3	8 (10.5%)
	Unknown	18 (23.7%)
Histological type	Ductal/no-special type	68 (89.5%)
	Lobular	4 (9.2%)
	Unknown	4 (9.2%)
ER [%]	Mean (SD)	87.53 (21.18)
	Median (quartiles)	100 (80–100)
	Range	10–100
PR [%]	Mean (SD)	58.54 (34.42)
	Median (quartiles)	70 (30–90)
	Range	1–100
HER2	Negative	40 (52.6%)
	Low **	33 (43.4%)
	Unknown (negative)	3 (4.0%)
Ki67 [%]	Mean (SD)	28.2 (13.76)
	Median (quartiles)	25 (17–35)
	Range	7–80
Visceral metastases	No	28 (36.8%)
	Yes	47 (61.8%)
	Unknown	1 (1.3%)
Brain metastases	No	72 (94.7%)
	Yes	4 (5.3%)
Bone metastases	No	25 (32.9%)
	Yes	51 (67.1%)
Performance status ***	0	8 (10.5%)
	1	22 (28.9%)
	2	22 (28.9%)
	3	17 (22.4%)
	4	2 (2.6%)
	Unknown	5 (6.6%)

* According to 8th edition American Joint Commission on Cancer classification. ** Immunohistochemistry scores of 1+ and 2+ without amplification (as determined by the in situ hybridization test). *** During the last visit. Abbreviations: ER, estrogen receptor; HER2, Human epidermal growth factor receptor 2; PR, progesterone receptor; SD, standard deviation.

**Table 2:** Previous treatment received.

Parameter		Total (N = 76)
Number of hormonal therapy lines in palliative treatment *	Mean (SD)	1.8 (0.83)
	Median (quartiles)	2 (1–2)
	Range	1–5
	n	76
Number of chemotherapy lines in palliative treatment	Mean (SD)	0.95 (0.98)
	Median (quartiles)	1 (0–2)
	Range	0–3
	n	76
Number of systemic therapy lines in palliative treatment	Mean (SD)	3.25 (1.24)
	Median (quartiles)	3 (2–4)
	Range	2–6
	n	76
Perioperative chemotherapy	No	31 (40.79%)
	Yes	45 (59.21%)
Chemotherapy during palliative treatment	No	32 (42.11%)
	Yes	44 (57.89%)
Any chemotherapy in the past	No	13 (17.11%)
	Yes	63 (82.89%)
Surgery for primary breast tumor	No	28 (36.84%)
	Yes	48 (63.16%)

Abbreviation: SD, standard deviation. * With or without targeted therapies such as cyclin-dependent kinase 4/6 inhibitors or alpelisib.

**Table 3:** Treatment received on the last visit.

Parameter		Total (N = 76)
Active treatment on the last visit	No treatment *	19 (25.0%)
	Chemotherapy	17 (22.4%)
	Hormonal therapy **	40 (52.6%)
Palliative care applied before the last visit	No	57 (75.0%)
	Yes	19 (25.0%)

* Including 6 patients who had their treatment ceased during the last visit with no intention to continue in the future. ** Including patients who received CDK4/6 inhibitors or alpelisib together with hormonal agents.

**Table 4:** Time from the last oncology visit to patients’ death.

Time from Last Visit to Death [Months]								
N	Missing	Mean	SD	Median	Min	Max	Q1	Q3
76	0	1.19	1.72	0.77	0	13.44	0.36	1.41

Abbreviations: SD, standard deviation; Q1, lower quartile; Q3, upper quartile.

## Data Availability

Data available on reasonable request.

## Supplementary Materials

The following supporting information can be downloaded at https://www.mdpi.com/article/10.3390/jcm13226739/s1: Table S1: Time from last visit to death and age at last visit; Table S2: Time from last visit to death and stage at diagnosis; Table S3: Time from last visit to death and tumor grade; Table S4: Time from last visit to death and estrogen receptor status; Table S5: Time from last visit to death and progesterone receptor status; Table S6: Time from last visit to death and human epidermal growth factor receptor 2 status; Table S7: Time from last visit to death and Ki67 level; Table S8: Time from last visit to death and type of systemic treatment at the last visit; Table S9: Time from last visit to death and application of palliative care; Table S10: Time from last visit to death and history of chemotherapy application for palliative systemic treatment; Table S11: Time from last visit to death and number of hormonal therapy lines in palliative systemic treatment; Table S12: Time from last visit to death and number of chemotherapy lines in palliative systemic treatment; Table S13: Time from last visit to death and number of chemotherapy lines in palliative systemic treatment; Table S14: Time from last visit to death and history of perioperative chemotherapy application; Table S15: Time from last visit to death and history of chemotherapy application in any setting; Table S16: Time from last visit to death and history of breast surgery; Table S17: Time from last visit to death and time from initial diagnosis to the last visit; Table S18: Time from last visit to death and time from metastatic disease diagnosis to the last visit; Table S19: Time from last visit to death and visceral metastases presence; Table S20: Time from last visit to death and CNS metastases presence.

## References

[1] Siegel R.L., Miller K.D., Fuchs H.E., Jemal A. (2022). Cancer Statistics, 2022. CA Cancer J. Clin..

[2] Burstein H.J., Curigliano G., Thürlimann B., Weber W.P., Poortmans P., Regan M.M., Senn H.J., Winer E.P., Gnant M., Aebi S. (2021). Customizing Local and Systemic Therapies for Women with Early Breast Cancer: The St. Gallen International Consensus Guidelines for Treatment of Early Breast Cancer 2021. Ann. Oncol..

[3] Cardoso F., Paluch-Shimon S., Senkus E., Curigliano G., Aapro M.S., André F., Barrios C.H., Bergh J., Bhattacharyya G.S., Biganzoli L. (2020). 5th ESO-ESMO International Consensus Guidelines for Advanced Breast Cancer (ABC 5). Ann. Oncol..

[4] Rashmi Kumar N., Schonfeld R., Gradishar W.J., Lurie R.H., Moran M.S., Abraham J., Abramson V., Aft R., Agnese D., Allison K.H. (2024). NCCN Guidelines Version 2.2024 Breast Cancer. https://www.nccn.org/professionals/physician_gls/pdf/breast.pdf.

[5] Cristofanilli M., Turner N.C., Bondarenko I., Ro J., Im S.A., Masuda N., Colleoni M., DeMichele A., Loi S., Verma S. (2016). Fulvestrant plus Palbociclib versus Fulvestrant plus Placebo for Treatment of Hormone-Receptor-Positive, HER2-Negative Metastatic Breast Cancer That Progressed on Previous Endocrine Therapy (PALOMA-3): Final Analysis of the Multicentre, Double-Blind, Phase 3 Randomised Controlled Trial. Lancet Oncol..

[6] Hortobagyi G.N., Stemmer S.M., Burris H.A., Yap Y.S., Sonke G.S., Paluch-Shimon S., Campone M., Petrakova K., Blackwell K.L., Winer E.P. (2018). Updated Results from MONALEESA-2, a Phase III Trial of First-Line Ribociclib plus Letrozole versus Placebo plus Letrozole in Hormone Receptor-Positive, HER2-Negative Advanced Breast Cancer. Ann. Oncol..

[7] Goetz M.P., Toi M., Campone M., Trédan O., Bourayou N., Sohn J., Park I.H., Paluch-Shimon S., Huober J., Chen S.C. (2017). MONARCH 3: Abemaciclib as Initial Therapy for Advanced Breast Cancer. J. Clin. Oncol..

[8] Rugo H.S., Finn R.S., Diéras V., Ettl J., Lipatov O., Joy A.A., Harbeck N., Castrellon A., Iyer S., Lu D.R. (2019). Palbociclib plus Letrozole as First-Line Therapy in Estrogen Receptor-Positive/Human Epidermal Growth Factor Receptor 2-Negative Advanced Breast Cancer with Extended Follow-Up. Breast Cancer Res. Treat..

[9] Sledge G.W., Toi M., Neven P., Sohn J., Inoue K., Pivot X., Burdaeva O., Okera M., Masuda N., Kaufman P.A. (2020). The Effect of Abemaciclib Plus Fulvestrant on Overall Survival in Hormone Receptor-Positive, ERBB2-Negative Breast Cancer That Progressed on Endocrine Therapy-MONARCH 2: A Randomized Clinical Trial. JAMA Oncol..

[10] Slamon D.J., Neven P., Chia S., Fasching P.A., De Laurentiis M., Im S.-A., Petrakova K., Bianchi G.V., Esteva F.J., Martín M. (2020). Overall Survival with Ribociclib plus Fulvestrant in Advanced Breast Cancer. N. Engl. J. Med..

[11] André F., Ciruelos E.M., Juric D., Loibl S., Campone M., Mayer I.A., Rubovszky G., Yamashita T., Kaufman B., Lu Y.S. (2021). Alpelisib plus Fulvestrant for PIK3CA-Mutated, Hormone Receptor-Positive, Human Epidermal Growth Factor Receptor-2-Negative Advanced Breast Cancer: Final Overall Survival Results from SOLAR-1. Ann. Oncol..

[12] Rugo H.S., Lerebours F., Ciruelos E., Drullinsky P., Borrego M.R., Neven P., Park Y.H., Prat A., Bachelot T., Juric D. (2020). Alpelisib (ALP) + Fulvestrant (FUL) in Patients (Pts) with PIK3CA-Mutated (Mut) Hormone Receptor-Positive (HR+), Human Epidermal Growth Factor Receptor 2-Negative (HER2–) Advanced Breast Cancer (ABC) Previously Treated with Cyclin-Dependent Kinase 4/6 Inhibitor (CDKi) + Aromatase Inhibitor (AI): BYLieve Study Results. J. Clin. Oncol..

[13] Beaver J.A., Park B.H. (2012). The BOLERO-2 Trial: The Addition of Everolimus to Exemestane in the Treatment of Postmenopausal Hormone Receptor-Positive Advanced Breast Cancer. Future Oncol..

[14] Bidard F.C., Kaklamani V.G., Neven P., Streich G., Montero A.J., Forget F., Mouret-Reynier M.A., Sohn J.H., Taylor D., Harnden K.K. (2022). Elacestrant (Oral Selective Estrogen Receptor Degrader) Versus Standard Endocrine Therapy for Estrogen Receptor-Positive, Human Epidermal Growth Factor Receptor 2-Negative Advanced Breast Cancer: Results from the Randomized Phase III EMERALD Trial. J. Clin. Oncol..

[15] Robson M.E., Im S.A., Senkus E., Xu B., Domchek S.M., Masuda N., Delaloge S., Tung N., Armstrong A., Dymond M. (2023). OlympiAD Extended Follow-up for Overall Survival and Safety: Olaparib versus Chemotherapy Treatment of Physician’s Choice in Patients with a Germline BRCA Mutation and HER2-Negative Metastatic Breast Cancer. Eur. J. Cancer.

[16] Litton J.K., Hurvitz S.A., Mina L.A., Rugo H.S., Lee K.H., Gonçalves A., Diab S., Woodward N., Goodwin A., Yerushalmi R. (2020). Talazoparib versus Chemotherapy in Patients with Germline BRCA1/2-Mutated HER2-Negative Advanced Breast Cancer: Final Overall Survival Results from the EMBRACA Trial. Ann. Oncol..

[17] Modi S., Saura C., Yamashita T., Park Y.H., Kim S.-B., Tamura K., Andre F., Iwata H., Ito Y., Tsurutani J. (2020). Trastuzumab Deruxtecan in Previously Treated HER2-Positive Breast Cancer. N. Engl. J. Med..

[18] Rugo H.S., Bardia A., Tolaney S.M., Arteaga C., Cortes J., Sohn J., Marmé F., Hong Q., Delaney R.J., Hafeez A. (2020). TROPiCS-02: A Phase III Study Investigating Sacituzumab Govitecan in the Treatment of HR+/HER2-Metastatic Breast Cancer. Future Oncol..

[19] Cherny N.I., Paluch-Shimon S., Berner-Wygoda Y. (2018). Palliative Care: Needs of Advanced Breast Cancer Patients. Breast Cancer Targets Ther..

[20] Telles A.C., de Souza São Bento P.A., Chagas M.C., de Queiroz A.B.A., de Mendonça Bittencourt N.C.C., da Silva M.M. (2021). Transition to Exclusive Palliative Care for Women with Breast Cancer. Rev. Bras. Enferm..

[21] Greer J.A., Moy B., El-Jawahri A., Jackson V.A., Kamdar M., Jacobsen J., Lindvall C., Shin J.A., Rinaldi S., Carlson H.A. (2022). Randomized Trial of a Palliative Care Intervention to Improve End-of-Life Care Discussions in Patients with Metastatic Breast Cancer. JNCCN J. Natl. Compr. Cancer Netw..

[22] Paiva C.E., Paiva B.S.R., Menezes D., Zanini L.E., Ciorlia J.B., Miwa M.U., Hui D. (2020). Development of a Screening Tool to Improve the Referral of Patients with Breast and Gynecological Cancer to Outpatient Palliative Care. Gynecol. Oncol..

[23] Sunilkumar M.M., Finni C.G., Lijimol A.S., Rajagopal M.R. (2021). Health-Related Suffering and Palliative Care in Breast Cancer. Curr. Breast Cancer Rep..

[24] Sarradon-Eck A., Besle S., Troian J., Capodano G., Mancini J. (2019). Understanding the Barriers to Introducing Early Palliative Care for Patients with Advanced Cancer: A Qualitative Study. J. Palliat. Med..

[25] Adamowicz K., Baczkowska-Waliszewska Z. (2020). Quality of Life During Chemotherapy, Hormonotherapy or AntiHER2 Therapy of Patients with Advanced, Metastatic Breast Cancer in Clinical Practice. Health Qual. Life Outcomes.

[26] Rabow M., Small R., Jow A., Majure M., Chien A., Melisko M., Belkora J., Esserman L.J., Rugo H. (2018). The Value of Embedding: Integrated Palliative Care for Patients with Metastatic Breast Cancer. Breast Cancer Res. Treat..

[27] Lucchi E., Berger F., Milder M., Commer J.M., Morin S., Capodano G., Thomaso M., Fogliarini A., Bremaud N., Henry A. (2024). Palliative Care Interventions and End-of-Life Care for Patients with Metastatic Breast Cancer: A Multicentre Analysis. Oncologist.

[28] Wolff A.C., Hammond M.E.H., Hicks D.G., Dowsett M., McShane L.M., Allison K.H., Allred D.C., Bartlett J.M.S., Bilous M., Fitzgibbons P. (2014). Recommendations for Human Epidermal Growth Factor Receptor 2 Testing in Breast Cancer: American Society of Clinical Oncology/College of American Pathologists Clinical Practice Guideline Update. Arch. Pathol. Lab. Med..

[29] Hammond M.E.H., Hayes D.F., Dowsett M., Allred D.C., Hagerty K.L., Badve S., Fitzgibbons P.L., Francis G., Goldstein N.S., Hayes M. (2010). American Society of Clinical Oncology/College of American Pathologists Guideline Recommendations for Immunohistochemical Testing of Estrogen and Progesterone Receptors in Breast Cancer. J. Clin. Oncol..

[30] Crawford G.B., Dzierżanowski T., Hauser K., Larkin P., Luque-Blanco A.I., Murphy I., Puchalski C.M., Ripamonti C.I. (2021). Care of the Adult Cancer Patient at the End of Life: ESMO Clinical Practice Guidelines. ESMO Open.

[31] Geyer T., Le N.S., Groissenberger I., Jutz F., Tschurlovich L., Kreye G. (2023). Systemic Anticancer Treatment Near the End of Life: A Narrative Literature Review. Curr. Treat. Options Oncol..

[32] Osei E., Lee D.-H., Kim S.Y. (2022). Comparison of Legislation, Regulations, and National Health Policies for Palliative Care in Three East Asian Countries: A Review of the Literature. J. Glob. Health Sci..

[33] van Gurp J., van Wijngaarden J., Payne S., Radbruch L., van Beek K., Csikós Á., Herder-Van der Eerden M., Hasselaar J. (2022). Integrating Palliative Care by Virtue of Diplomacy; A Cross-Sectional Group Interview Study of the Roles and Attitudes of Palliative Care Professionals to Further Integrate Palliative Care in Europe. Int. J. Health Policy Manag..

[34] Clark D., Baur N., Clelland D., Garralda E., López-Fidalgo J., Connor S., Centeno C. (2020). Mapping Levels of Palliative Care Development in 198 Countries: The Situation in 2017. J. Pain Symptom Manag..

[35] Beaudet M.É., Lacasse Y., Labbé C. (2022). Palliative Systemic Therapy Given near the End of Life for Metastatic Non-Small Cell Lung Cancer. Curr. Oncol..

[36] Schnipper L.E., Smith T.J., Raghavan D., Blayney D.W., Ganz P.A., Mulvey T.M., Wollins D.S. (2012). American Society of Clinical Oncology Identifies Five Key Opportunities to Improve Care and Reduce Costs: The Top Five List for Oncology. J. Clin. Oncol..

[37] Rugo H.S., Bardia A., Marmé F., Cortés J., Schmid P., Loirat D., Trédan O., Ciruelos E., Dalenc F., Gómez Pardo P. (2023). Overall Survival with Sacituzumab Govitecan in Hormone Receptor-Positive and Human Epidermal Growth Factor Receptor 2-Negative Metastatic Breast Cancer (TROPiCS-02): A Randomised, Open-Label, Multicentre, Phase 3 Trial. Lancet.

[38] Modi S., Jacot W., Yamashita T., Sohn J., Vidal M., Tokunaga E., Tsurutani J., Ueno N.T., Prat A., Chae Y.S. (2022). Trastuzumab Deruxtecan in Previously Treated HER2-Low Advanced Breast Cancer. N. Engl. J. Med..

[39] Bardia A., Krop I.E., Kogawa T., Juric D., Tolcher A.W., Hamilton E.P., Mukohara T., Lisberg A., Shimizu T., Spira A.I. (2024). Datopotamab Deruxtecan in Advanced or Metastatic HR+/HER2- and Triple-Negative Breast Cancer: Results From the Phase I TROPION-PanTumor01 Study. J. Clin. Oncol..

[40] ECOG Performance Status Scale—ECOG-ACRIN Cancer Research Group. https://ecog-acrin.org/resources/ecog-performance-status/.

[41] Prigerson H.G., Bao Y., Shah M.A., Elizabeth Paulk M., LeBlanc T.W., Schneider B.J., Garrido M.M., Carrington Reid M., Berlin D.A., Adelson K.B. (2015). Chemotherapy Use, Performance Status, and Quality of Life at the End of Life. JAMA Oncol..

[42] Püsküllüoğlu M., Pieniążek M., Rudzińska A., Pietruszka A., Pacholczak-Madej R., Grela-Wojewoda A., Ziobro M. (2024). Cisplatin Monotherapy as a Treatment Option for Patients with HER-2 Negative Breast Cancer Experiencing Hepatic Visceral Crisis or Impending Visceral Crisis. Oncol. Ther..

[43] Rochigneux P., Raoul J.L., Beaussant Y., Aubry R., Goldwasser F., Tournigand C., Morin L. (2017). Use of Chemotherapy near the End of Life: What Factors Matter?. Ann. Oncol..

[44] Temel J.S., Greer J.A., Muzikansky A., Gallagher E.R., Admane S., Jackson V.A., Dahlin C.M., Blinderman C.D., Jacobsen J., Pirl W.F. (2010). Early Palliative Care for Patients with Metastatic Non-Small-Cell Lung Cancer. N. Engl. J. Med..

[45] Smith T.J., Temin S., Alesi E.R., Abernethy A.P., Balboni T.A., Basch E.M., Ferrell B.R., Loscalzo M., Meier D.E., Paice J.A. (2012). American Society of Clinical Oncology Provisional Clinical Opinion: The Integration of Palliative Care into Standard Oncology Care. J. Clin. Oncol..

[46] Ziegler L.E., Craigs C.L., West R.M., Carder P., Hurlow A., Millares-Martin P., Hall G., Bennett M.I. (2018). Is Palliative Care Support Associated with Better Quality End-of-Life Care Indicators for Patients with Advanced Cancer? A Retrospective Cohort Study. BMJ Open.

[47] Schultz M., Baziliansky S., Mitnik I., Ulitzur N., Illouz S., Katra D., Givoli S., Campisi-Pinto S., Bar-Sela G., Zalman D. (2023). Associations Between Psycho-Social-Spiritual Interventions, Fewer Aggressive End-of-Life Measures, and Increased Time After Final Oncologic Treatment. Oncologist.

[48] Woldie I., Elfiki T., Kulkarni S., Springer C., McArthur E., Freeman N. (2022). Chemotherapy During the Last 30 Days of Life and the Role of Palliative Care Referral, a Single Center Experience. BMC Palliat. Care.

