# Supplementary material for: Time from Final Oncologist Visit to Death and Palliative Systemic Treatment Use Near the End of Life in Heavily Pretreated Patients with Luminal Breast Cancer
Source: J Clin Med. 2024 Nov 8;13(22):6739. doi: 10.3390/jcm13226739 (PMC11594325; doi:10.3390/jcm13226739)
Supplement: Supplementary file 1 [file jcm-13-06739-s001.zip › jcm-3286226-supplementary.pdf]

Supplementary Materials

Table S1. Time from last visit to death and age at last visit

Variables	Spearman's correlation coefficient	p
Age at the last visit & **time** from last visit to death	0.058	p=0.618

Abbreviation: p - p-value (statistical significance)

Table S2. Time from last visit to death and stage at diagnosis

Stage at diagnosis	N	Time from last visit to death [months]							p
		Mean	SD	Median	Min	Max	Q1	Q3	
Metastase de novo	19	1,18	1,17	0,89	0	4,07	0,44	1,22	p=0.471
Radical treatment	55	1,22	1,90	0,72	0	13,44	0,36	1,51	
Locally advanced breast cancer	*2*	*0,39*	*0,56*	*0,39*	*0*	*0,79*	*0,20*	*0,59*	

Abbreviations: p - Kruskal-Wallis test, SD - standard deviation, Q1 - lower quartile, Q3 - upper quartile

Table S3. Time from last visit to death and tumor grade

Grade	N	Time from last visit to death [months]							p
		Mean	SD	Median	Min	Max	Q1	Q3	
G1	3	2,65	1,46	1,87	1,74	4,34	1,81	3,10	p=0.073
G2	40	1,16	2,11	0,71	0,00	13,44	0,34	1,28	
G3	6	1,45	1,50	0,67	0,26	3,78	0,43	2,37	

Abbreviations: p - Kruskal-Wallis test, SD - standard deviation, Q1 - lower quartile, Q3 - upper quartile

Table S4. Time from last visit to death and estrogen receptor status

Variables	Spearman's correlation coefficient	p
ER & Time from last visit to death	-0.063	p=0.612

Abbreviation: p - p-value (statistical significance)

Table S5. Time from last visit to death and progesterone receptor status

Variables	Spearman's correlation coefficient	p
PR & Time from last visit to death	0.071	p=0.6

Abbreviation: p - p-value (statistical significance)

Table S6. Time from last visit to death and human epidermal growth factor receptor 2 status

HER-2	N	Time from last visit to death [months]							p
		Mean	SD	Median	Min	Max	Q1	Q3	
Negative	40	1,28	2,15	0,82	0,00	13,44	0,36	1,39	p=0.684
Positive	11	0,82	0,84	0,72	0,13	3,09	0,26	0,97	
No amplification	22	1,11	1,14	0,71	0,00	4,07	0,43	1,63	

Abbreviations: HER-2 - human epidermal growth factor receptor 2 status, p - Kruskal-Wallis test, SD - standard deviation, Q1 - lower quartile, Q3 - upper quartile

Table S7. Time from last visit to death and Ki67 level

Variables	Spearman's correlation coefficient	p
Ki67 & Time from last visit to death	0.09	p=0.487

Abbreviation: p - p-value (statistical significance)

Table S8. Time from last visit to death and type of systemic treatment at the last visit

Active treatment during the last visit	N	Time from last visit to death [months]							p
		Mean	SD	Median	Min	Max	Q1	Q3	
No treatment	19	1,64	2,95	0,89	0,16	13,44	0,43	1,30	p=0.735
Chemotherapy	17	1,11	1,22	0,72	0,00	4,07	0,23	1,38	
Hormonotherapy	40	1,02	0,95	0,66	0,00	4,34	0,36	1,43	

Abbreviations*:* p - Kruskal-Wallis test, SD - standard deviation, Q1 - lower quartile, Q3 - upper quartile

Table S9. Time from last visit to death and application of palliative care

In home-base hospice or outpatient palliative cae at the last visit	N	Time from last visit to death [months]							p
		Mean	SD	Median	Min	Max	Q1	Q3	
No	57	1,34	1,94	0,79	0,00	13,44	0,36	1,48	p=0.272
Yes	19	0,74	0,53	0,53	0,03	1,81	0,36	1,07	

Abbreviations*:* p - Mann-Whitney test, SD - standard deviation, Q1 - lower quartile, Q3 - upper quartile

Table S10. Time from last visit to death and history of chemotherapy application for palliative systemic treatment

Chemotherapy during palliative treatment	N	Time from last visit to death [months]							p
		Mean	SD	Median	Min	Max	Q1	Q3	
No	41	0,96	0,97	0,56	0	4,34	0,36	1,25	p=0.283
Yes	35	1,47	2,29	0,85	0	13,44	0,43	1,63	

Abbreviations*:* p - Mann-Whitney test, SD - standard deviation, Q1 - lower quartile, Q3 - upper quartile

Table S11. Time from last visit to death and number of hormonal therapy lines in palliative systemic treatment.

Variables	Spearman's correlation coefficient	p
Hormonotherapy lines in palliative treatment & Time from last visit to death	0.197	p=0.089

Abbreviation: p - p-value (statistical significance)

Table S12. Time from last visit to death and number of chemotherapy lines in palliative systemic treatment.

Variables	Spearman's correlation coefficient	p
Chemotherapy lines during palliative treatment & Time from last visit to death	0.073	p=0.53

Abbreviation: p - p-value (statistical significance)

Table S13. Time from last visit to death and number of chemotherapy lines in palliative systemic treatment.

Variables	Spearman's correlation coefficient	p
Therapy lines during paliative treatment & Time from last visit to death	0.196	p=0.09

Abbreviation: p - p-value (statistical significance)

Table S14. Time from last visit to death and history of perioperative chemotherapy application.

Perioperative chemotherapy	N	Time from last visit to death [months]							p
		Mean	SD	Median	Min	Max	Q1	Q3	
No	31	1,09	1,11	0,85	0	4,07	0,36	1,33	p=0.796
Yes	45	1,26	2,04	0,76	0	13,44	0,43	1,41	

Abbreviations: p - Mann-Whitney test, SD - standard deviation, Q1 - lower quartile, Q3 - upper quartile

Table S15. Time from last visit to death and history of chemotherapy application in any setting.

Chemotherapy in any setting	N	Time from last visit to death [months]							p
		Mean	SD	Median	Min	Max	Q1	Q3	
No	13	1,04	1,03	0,53	0,23	3,78	0,36	1,25	p=0.868
Yes	63	1,22	1,83	0,79	0,00	13,44	0,38	1,41	

Abbreviations: p - Mann-Whitney test, SD - standard deviation, Q1 - lower quartile, Q3 - upper quartile

Table S16. Time from last visit to death and history of breast surgery.

Surgery	N	Time from last visit to death [months]							p
		Mean	SD	Median	Min	Max	Q1	Q3	
No	28	1,17	1,04	0,90	0	4,07	0,45	1,41	p=0.294
Yes	48	1,20	2,02	0,59	0	13,44	0,34	1,43	

Abbreviations: p - Mann-Whitney test, SD - standard deviation, Q1 - lower quartile, Q3 - upper quartile

Table S17. Time from last visit to death and time from initial diagnosis to the last visit.

Variables	Spearman's correlation coefficient	p
Time from diagnosis to the last visit & Time from last visit to death	-0.118	p=0.312

Abbreviation: p - p-value (statistical significance)

Table S18. Time from last visit to death and time from metastatic disease diagnosis to the last visit.

Variables	Spearman's correlation coefficient	p
Time from metastases diagnosis to the last visit & Time from last visit to death	0.09	p=0.437

Abbreviation: p - p-value (statistical significance)

Table S19. Time from last visit to death and visceral metastases presence.

Visceral metastases	N	Time from last visit to death [months]							p
		Mean	SD	Median	Min	Max	Q1	Q3	
No	28	0,98	0,98	0,59	0	4,34	0,36	1,41	p=0.565
Yes	47	1,34	2,04	0,79	0	13,44	0,38	1,56	

Abbreviations: p - Mann-Whitney test, SD - standard deviation, Q1 - lower quartile, Q3 - upper quartile

Table S20 Time from last visit to death and CNS metastases presence.

CNS metastases	N	Time from last visit to death [months]							p
		Mean	SD	Median	Min	Max	Q1	Q3	
No	72	1,23	1,76	0,79	0,00	13,44	0,36	1,43	p=0.415
Yes	4	0,59	0,51	0,54	0,03	1,25	0,33	0,80	

Abbreviations: CNS – central nervous system, p - Mann-Whitney test, SD - standard deviation, Q1 - lower quartile, Q3 - upper quartile
